# Efficacy of Physical Exercise on the Quality of Life, Exercise Ability, and Cardiopulmonary Fitness of Patients With Atrial Fibrillation: A Systematic Review and Meta-Analysis

**DOI:** 10.3389/fphys.2020.00740

**Published:** 2020-07-24

**Authors:** Shuqing Shi, Jingjing Shi, Qiulei Jia, Shuai Shi, Guozhen Yuan, Yuanhui Hu

**Affiliations:** ^1^Department of Cardiovascular, Guang'anmen Hospital, China Academy of Chinese Medical Sciences, Beijing, China; ^2^Department of Clinical Medicine, Beijing University of Chinese Medicine, Beijing, China

**Keywords:** physical exercise, atrial fibrillation, meta-analysis, randomized controlled trials, quality of life

## Abstract

**Objective:** Physical exercise is recommended to help prevent lifestyle diseases. The present study was designed to quantify the efficacy of physical exercise on the quality of life (QoL), exercise ability and cardiopulmonary fitness of patients with atrial fibrillation (AF).

**Method:** A comprehensive systematic literature search was performed in Medline, Embase, Cochrane Library, Web of Science and PubMed databases (from 1970 to December 1st, 2019) for randomized controlled trials (RCTs) comparing physical exercise combined with AF routine treatments to routine treatments alone. The meta-analysis was conducted following PRISMA guidelines. Our main outcomes were QoL (measured by the Short-Form 36 scale, SF-36), exercise ability (measured by the 6-min walk test, 6MWT) and cardiopulmonary fitness (measured by peak oxygen uptake and resting heart rate). Quality assessments were conducted using the Cochrane Collaboration tool.

**Results:** Twelve trials involving 819 patients met the criteria for analysis. The results showed that physical exercise improved the QoL by enhancing physical functioning [standardized mean difference (SMD) = 0.63, 95%CI: 0.18–1.09; *p* = 0.006], general health perceptions (SMD = 0.64, 95%CI: 0.35–0.93; *p* < 0.001) and vitality (SMD = 0.51, 95%CI: 0.31–0.71; *p* < 0.001); increased exercise ability by improving the 6MWT performance (SMD = 0.69, 95%CI: 0.19–1.119; *p* = 0.007); and enhanced peak VO_2_ (SMD = 0.37, 95%CI: 0.16–0.57; *p* < 0.001) while reducing resting heart rate (SMD = −0.39, 95%CI: −0.65 to −0.13; *p* = 0.004). In addition, meta-regression analysis showed that training mode (*p*_physicalfunctioning_ = 0.012, *p*_generalhealthperceptions_ = 0.035) and training duration (*p* = 0.047) were the main factors of an intervention that influenced the effect size. Following sub-group analysis, we found that aerobics, Yoga and longer training durations (≥60 min) showed larger improvements.

**Conclusion:** In summary, our meta-analysis shows that physical exercise has a positive effect on the QoL, exercise ability and cardiopulmonary fitness in AF patients. When physicians offer exercise recommendations to AF patients, they should consider both the training mode and training duration to achieve maximum results.

## Introduction

Atrial fibrillation (AF) is the most common cardiac arrhythmia characterized by the high-frequency electrical activity of the atrium, unsynchronized atrial contraction and irregular ventricular excitation (Calkins et al., 2017). In 2010, there were 20.9 million males and 12.6 million females who suffered from AF worldwide (Chugh et al., 2014). As a global pandemic, the number of AF patients is continuously increasing and is estimated to rise to 12.1 million in the United States by 2030 (Morin et al., 2016) and 16.9 million in the European Union by 2060 (Krijthe et al., 2013).

The high incidence of AF is associated with increased rates of mortality and disability. The risk of stroke is 4 to 5 times higher in AF than non-AF patients, leading to a mortality rate of nearly 20% and disability rate of almost 60% (Ganesan et al., 2016; Hahne et al., 2016). In addition, AF causes high mortality rates because of sudden cardiac death (Ruwald et al., 2013; Ohsawa et al., 2015). Due to its prevalence and potentially serious complications, the social burden and economic pressure caused by AF should not be ignored in either developed or developing countries. Approximately 50–70% of the financial burden of AF is attributable to hospitalization costs. In the USA, there was a 24% increase in the mean cost of AF hospitalization, with a corresponding increase in the economic burden by nearly $1.31 billion (Patel et al., 2014). It can be said that AF has become a major public health problem affecting the improvement of the national residents' health level and hindering social and economic development (Leong et al., 2017).

The main AF symptoms are fatigue, decreased exercise tolerance, dyspnea and palpitations, which lead to decreased QoL in 58% of cases (Randolph et al., 2016). Exercise capacity and cardiopulmonary fitness will also be reduced to varying degrees (Ariansen et al., 2011). Over the past decade, the focus of AF treatment has shifted from electrocardiograph results to symptom control (Pinter and Dorian, 2010). Physical exercise can effectively assist in the therapy of various types of cardiovascular diseases, and the potential benefits of moderate regular physical exercise in reducing the risk of AF are considerable (Eckel et al., 2014). Randomized studies of physical exercise in patients with AF found that significant improvements in QoL and exercise ability can be achieved through a short-term exercise training program (Hegbom et al., 2007; Osbak et al., 2012; Kato et al., 2019).

However, this is not always the case. Studies have revealed that vigorous physical activity, especially long-term endurance exercise, may lead to excessive heart adaptation and physiological changes, thereby increasing the risk of AF (Aizer et al., 2009; Thelle et al., 2013). Existing research indicates a wide range of potential mechanisms, most of which are still to be determined. Pelliccia et al. (2005) demonstrated that training-induced atrial enlargement was present in 20% of athletes, which is one of the major factors for atrial remodeling. This may be due to excessive exercise that increases the volume load of the atrium (Baggish and Wood, 2011). For leisure-time runners, whether the left atrium expands depends on the exercise load (Wilhelm, 2014). Myocardial damage shown by delayed-enhancement cardiac MRI imaging was prevalent in presumably healthy marathon runners and appeared to be associated with an increased coronary event rate (Breuckmann et al., 2009), which also subsequently leads to a higher rate of myocardial fibrosis and remodeling in asymptomatic veteran athletes (Wilson et al., 2011).

Three meta-analyses (Giacomantonio et al., 2013; Reed et al., 2013; Risom et al., 2017) and one review (Myrstad et al., 2019) have evaluated the effects of exercise on patients with AF. Risom et al. (2017) found that exercise-based rehabilitation for AF patients significantly increased their exercise capacity; however, the authors considered not only trials of exercise training but also trials that included a psycho-educational interventional component. Myrstad et al. (2019) reviewed studies that investigated the effects and safety of exercise in individuals with AF and provided individualized exercise advice for different types of AF patients (such as whether the patient has an exercise restriction, a health assessment is needed before exercise or medication is needed before exercise). However, although meta-analyses and reviews have been reported in this field, it is not clear whether the effect of exercise on AF patients is quantitatively related to specific exercise variables (pattern, duration and frequency), and considering the dual effects of exercise on AF onset, the impact of exercise on the frequency and severity of AF symptoms is still unknown. In addition, the previously reported meta-analysis needs to be updated as new RCTs have been published.

Therefore, the purpose of this meta-analysis was to provide an update and address the current gaps in this field. Specifically, we aimed to simultaneously clarify the effects of physical exercise on the QoL, physical exercise and cardiopulmonary fitness of AF patients and identify variables that influence the beneficial effects of exercise on AF patients. In addition, we clarified the effect of exercise on the onset of AF.

## Methods

This present meta-analysis was conducted following by the Preferred Reporting Items for Systematic Reviews and Meta-Analyses (PRISMA) guidelines (Shamseer et al., 2015). The data used in this study are all secondary and do not require ethical approval.

### Search Strategy

A systematic literature search was conducted in five electronic databases, including Medline, Embase, the Cochrane Library, Web of Science and PubMed, from 1970 to December 1st, 2019 for RCTs studying the effects of physical exercise on the QoL, exercise ability and cardiopulmonary fitness of patients with AF. Keywords and their medical subject headings (MeSH) or Embase subject headings (EMTREE) were used for the search strategy. Table 1 shows the details of the PubMed search strategy.

**Table 1:** PubMed search strategy.

**No**.	**Search items**
#1	(((((((((Atrial Fibrillations[Title/Abstract]) OR Fibrillation, Atrial[Title/Abstract]) OR Auricular Fibrillation[Title/Abstract]) OR Fibrillation, Auricular[Title/Abstract]) OR Persistent Atrial Fibrillation[Title/Abstract]) OR Familial Atrial Fibrillation [Title/Abstract]) OR Paroxysmal Atrial Fibrillation[Title/Abstract]) OR Atrial Fibrillation, Paroxysmal[Title/Abstract])) OR “Atrial Fibrillation”[Mesh]
#2	Search ((((((((((((((((((((Exercises[Title/Abstract]) OR Physical Activity[Title/Abstract]) OR Activities, Physical[Title/Abstract]) OR Activity, Physical[Title/Abstract]) OR Physical Activities[Title/Abstract]) OR Exercise, Physical[Title/Abstract]) OR Exercises, Physical[Title/Abstract]) OR Physical Exercise[Title/Abstract]) OR Physical Exercises[Title/Abstract]) OR Acute Exercise[Title/Abstract]) OR Acute Exercises[Title/Abstract]) OR Exercise, Acute[Title/Abstract]) OR Exercises, Acute[Title/Abstract]) OR Exercise, Isometric[Title/Abstract]) OR Exercises, Isometric[Title/Abstract]) OR Isometric Exercises[Title/Abstract]) OR Isometric Exercise[Title/Abstract]) OR Exercise, Aerobic[Title/Abstract]) OR Aerobic Exercise[Title/Abstract])) OR “Exercise”[Mesh]
#3	((randomized controlled trial [Publication Type]) OR randomized [Title/Abstract]) OR placebo [Title/Abstract]
#4	#1 AND #2 AND #3

### Inclusion Criteria

Eligible studies were identified if they met all the following criteria:

1. Population: adults with AF (aged 18 and over with no upper age limit).
2. Intervention: studies investigating the effects of physical exercise on the QoL, exercise ability and cardiopulmonary fitness of AF patients.
3. Comparison: treatment as usual (e.g., standard medical care, such as drug and ablation therapy).
4. Outcome(s): QoL measured by the SF-36 scale, exercise ability measured by the 6MWT, and cardiopulmonary fitness measured by peak oxygen uptake (peak VO_2_) and resting heart rate.
5. Study type: RCT.

### Exclusion Criteria

1. Animal studies, case reports, reviews, abstracts, conference proceedings and editorials.
2. Non-RCTs.
3. Studies without sufficient data to calculate SMD.

### Data Extraction

Microsoft Excel 2019 was used to set up the data extraction table. The main components of the extracted information were classified as:

1. Publication information: first author and publication year.
2. General characteristics of patients: sample size, gender and age.
3. Details of intervention and control therapy: training period, session duration, training mode and number of sessions per week.
4. Details of outcomes.
5. Bias risk assessment information: quality of included studies.

### Statistical Analyses

Stata SE software version 15.0 was used for all statistical analyses. We used the SMD with the corresponding 95% confidence intervals (CI) as the effect size (ES). If the data (including the sample size, mean value and standard deviation) extracted from the RCTs met the needs of the analyses, the analyses were conducted directly (Higgins et al., 2008a). If the data (including the standard error, 95%CI or *p*-values) in the RCTs could not be used directly for analyses, they were analyzed after data conversion (Higgins et al., 2008b; Morris, 2008; Guilera et al., 2013). Cochran's Q test and *I*^2^ test were used to evaluate heterogeneity. A *p*-value < 0.1 or *I*^2^ value > 50% indicated significant heterogeneity between RCTs, and the random-effect model was used. Otherwise, a fixed-effect model was used (Higgins et al., 2003). Meta-regression was used to identify the possible factors that may cause high heterogeneity, and subgroup analysis was conducted based on these factors. The changes in heterogeneity before and after subgroup analyses were compared. Begg's test and Egger's test were used to assess the publication bias, and a *p*-value < 0.1 indicated the presence of publication bias.

### Assessment of the Risk of Bias

The Cochrane Collaboration tool (Higgins et al., 2011) was used to assess the risk of bias. Two authors (SQS and JS) independently extracted data and assessed the quality of the included studies. The data were recorded on a special data form. The differences between data extraction and quality evaluation were determined through discussion.

## Results

### Search Results

**Figure 1:**
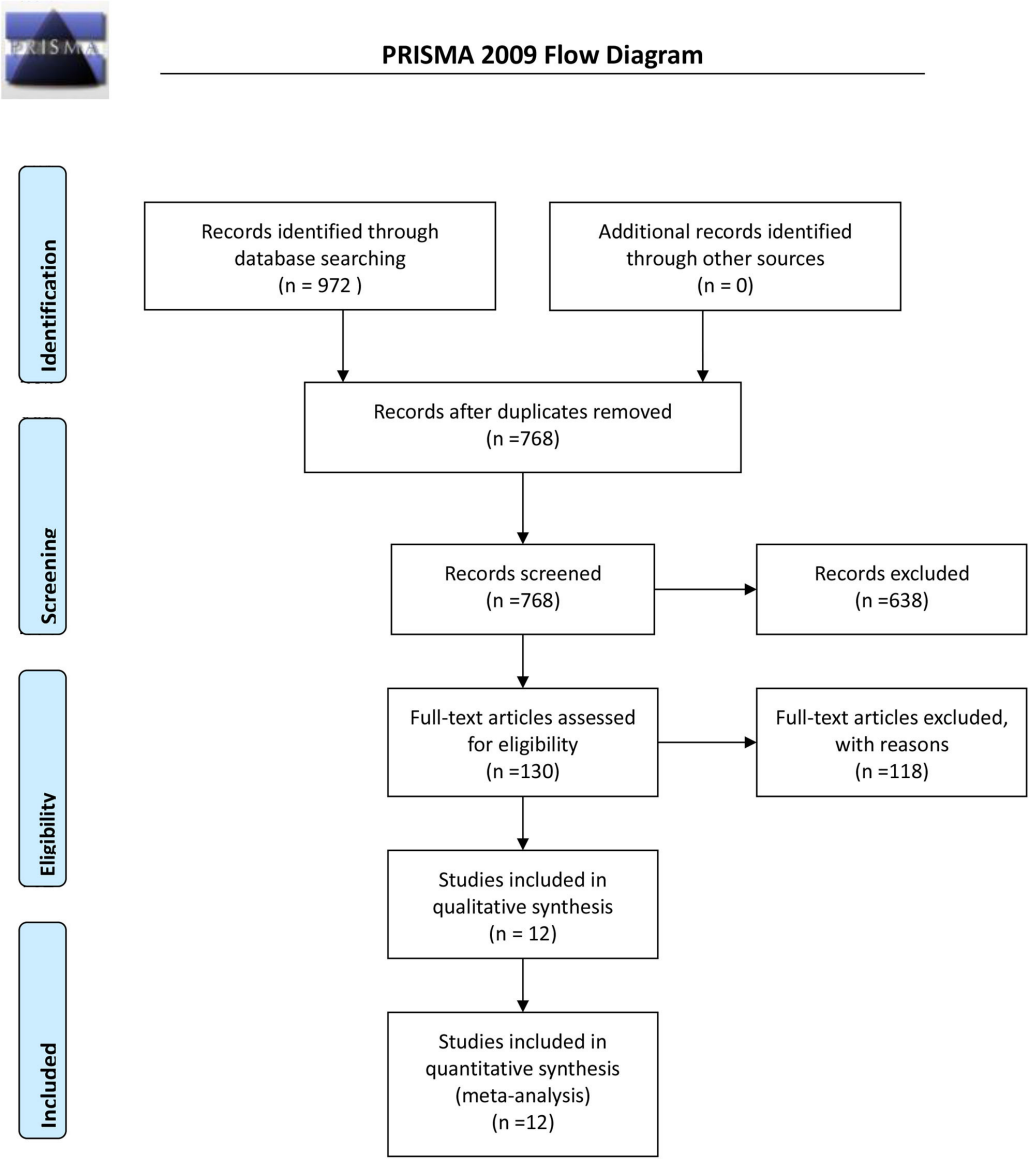
Flow diagram of the search procedure.

**Figure 2:**
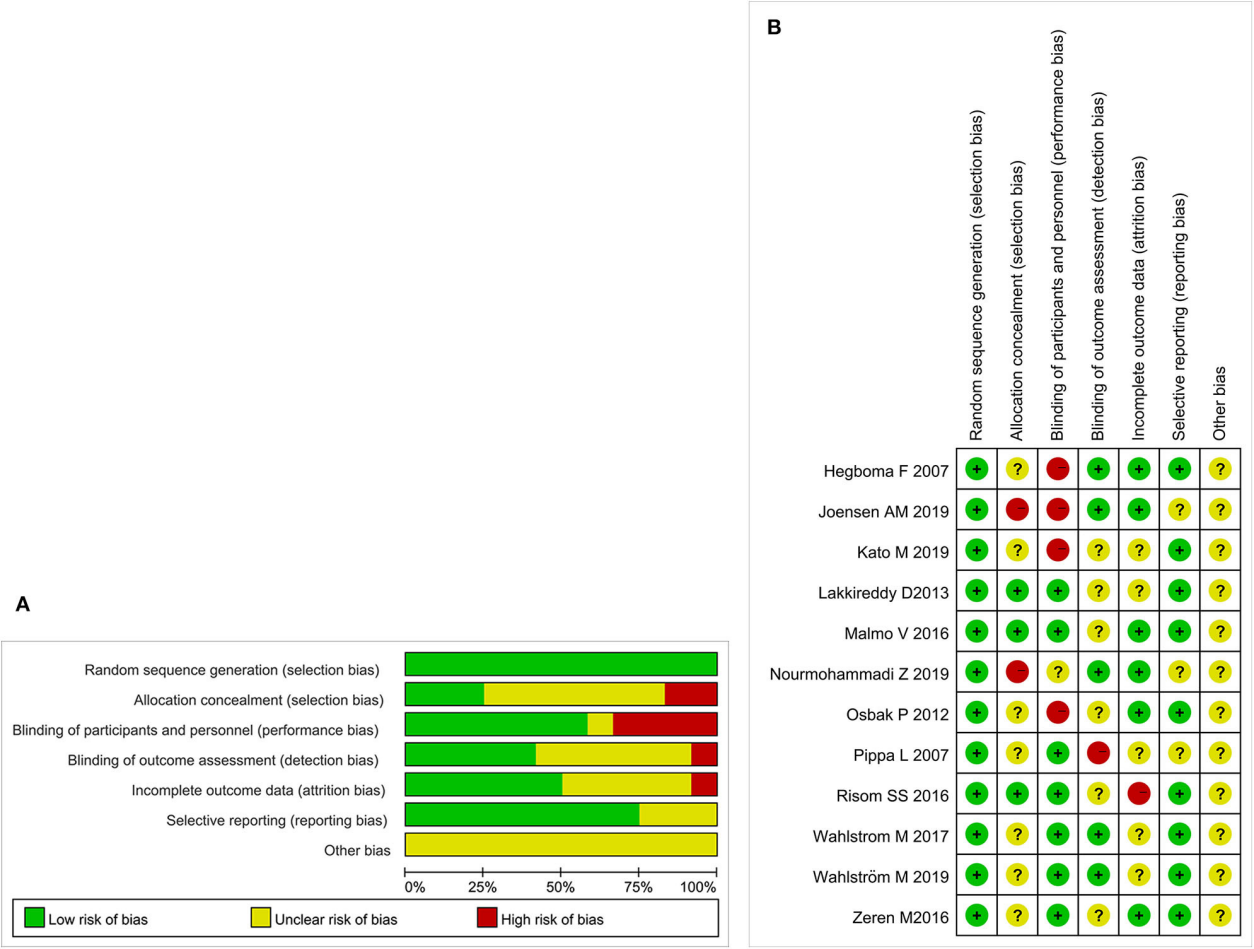
**(A)** The risk of bias. A review of the authors' judgments about each risk of bias items are presented as percentages across all of the included studies. The quality of the selected studies is assessed according to the Cochrane criteria. **(B)** The risk of bias summary. A review of the authors' judgments about the risk of bias are included in each study.

Initially, 972 potential studies were retrieved. After deleting duplicates, 768 studies were screened. After scanning the titles and abstracts, the inconsistencies were eliminated, and 130 papers remained. Following a careful reading of the full text using pre-established inclusion and exclusion criteria, 118 articles were excluded, and the remaining 12 were included in the meta-analysis. The flow chart of the search process is shown in Figure 1. The bias condition of selected studies is shown in Figures 2A,B.

### Trial and Patient Characteristics

The 12 RCTs included 819 patients (408 allocated to experimental groups and 411 allocated to control groups). Four RCT exercise interventions were aerobic (Osbak et al., 2012; Malmo et al., 2016; Joensen et al., 2019; Nourmohammadi et al., 2019), one was anaerobic (Zeren et al., 2016), three were aerobic combined with anaerobic (Hegbom et al., 2007; Risom et al., 2016; Kato et al., 2019), one was Qigong (Pippa et al., 2007), and three were Yoga (Lakkireddy et al., 2013; Wahlstrom et al., 2017; Wahlström et al., 2020). The details of all included studies are shown in Table 2. The classification and description of exercise interventions are presented in Table 3.

**Table 2:** Data extraction of the selected articles.

**Study**	**Country**	**Inclusion criteria**	**Case (*n*)**		**Male (*n*)**	**Training period**	**Training mode**	**Session duration**	**Number of sessions per week**	**Exercise intensity**	**Control measures**	**Primary outcomes**
			**FG**	**CG**								
Osbak et al. (2012)	Denmark	Permanent AF	24	23	35	12 weeks	Aerobics	60min	3 times	70% of max exercise capacity	AF conventional treatment	Muscle strength Exercise capacity (6MWT) Lean body mass Fat percentage QoL (SF-36)
Kato et al. (2019)	Japan	Persistent AF	28	31	48	6 months	Combination	30min	2–3 times	40%-60% of max exercise capacity	Usual care for AF	Exercise capacity (6MWT) Cardiac function (peak VO_2_, LAD) Inflammatory status (CRP, IL-6) safety
Joensen et al. (2019)	Denmark	Paroxysmal or persistent AF	28	24	34	12 weeks	Aerobics	60min	2 times	70% of max exercise capacity	AF usual treatment	QoL (AF-QoL-18, A FEQT, GAD-7, PHQ-9, EQ-5D) Physical exercise (6MWT, 5RSST)
Malmo et al. (2016)	Norway	Paroxysmal or persistent AF	26	25	42	12 weeks	Aerobics	45min	3 times	85–95% of max HR	AF usual treatment	Time in AF Cardiac function (peak VO_2_) AF symptoms QoL (SF-36), exercise capacity
Nourmohammadi et al. (2019)	Iran	Chronic AF	25	25	23	8 weeks	Aerobics	60min	2 times	70–80% of max HR	Routine care program	QoL (SF-36)
Zeren et al. (2016)	Turkey	Permanent AF	17	16	17	12 weeks	Anaerobic	30min	3 times	30% of max HR	Standard medical treatment only	6MWT
Hegbom et al. (2007)	Norway	Chronic AF	13	15	26	8 weeks	Combination	75min	3 times	70–90% of max HR	Routine care program	QoL (SF-36) The Symptoms and Severity Checklist (SSCL) Activities of Daily Living (ADL) Symptom Score (SS)
Risom et al. (2016)	Denmark	Paroxysmal or persistent AF.	105	105	151	12 weeks	Combination	NK	3 times	NK	Usual care.	Peak VO_2_ Max. blood pressure QoL (SF-36) Mortality Adverse events
Pippa et al. (2007)	Italy	Paroxysmal or persistent AF.	22	21	30	16 weeks	Qigong	90min	2 times	NK	Usual care.	6MWT
Lakkireddy et al. (2013)	America	Paroxysmal AF	49	49	46	12 weeks	Yoga	60min	2 times	NK	Usual care.	Change in symptomatic AF Symptomatic non-AF, asymptomatic AF episodes QoL (SF-36) Zung self-assessment anxiety score (SAS) Zung self-assessment depression score (SDS).
Wahlström et al. (2020)	Sweden	Paroxysmal AF	38	41	38	12 weeks	Yoga	60min	1 time	NK	Usual care.	QoL (SF-36) Blood pressure HR and NT-proBNP
Wahlstrom et al. (2017)	Sweden	Paroxysmal AF	33	36	42	12 weeks	Yoga	60min	1 time	NK	Usual care.	QoL (EQ-5D VAS, SF-36) Blood pressure HR

**Table 3:** Classification and description of exercise interventions.

**Exercise interventions**	**Description**
Aerobic	Exercise that involves repetitive movement of large muscle groups to improve cardiorespiratory endurance, usually performed at moderate to vigorous intensity for prolonged periods
Anaerobic	Exercise that uses an external resistance load (e.g., body weight or resistance bands) to improve the ability of skeletal muscles to exert force
Combination	Intervention combining aerobic and anaerobic exercises
Qigong	Physical and mental exercise aimed at strengthening the body, preventing and treating diseases, maintaining fitness and developing one's potential by adjusting breath, physical activity and consciousness
Yoga	Exercise that consists of a complex system of moral, spiritual and physical practices to attain self-awareness. These basic themes run through modern Western yoga with a focus on posture, muscle stretching, breathing exercises, and meditation

### Effects of Physical Exercise on QoL in Patients With AF

Based on the data from the included studies, we analyzed the score of physical functioning, general health perceptions and vitality in the SF-36 scale to measure the impact of exercise on the QoL of patients with AF.

**Figure 3:**
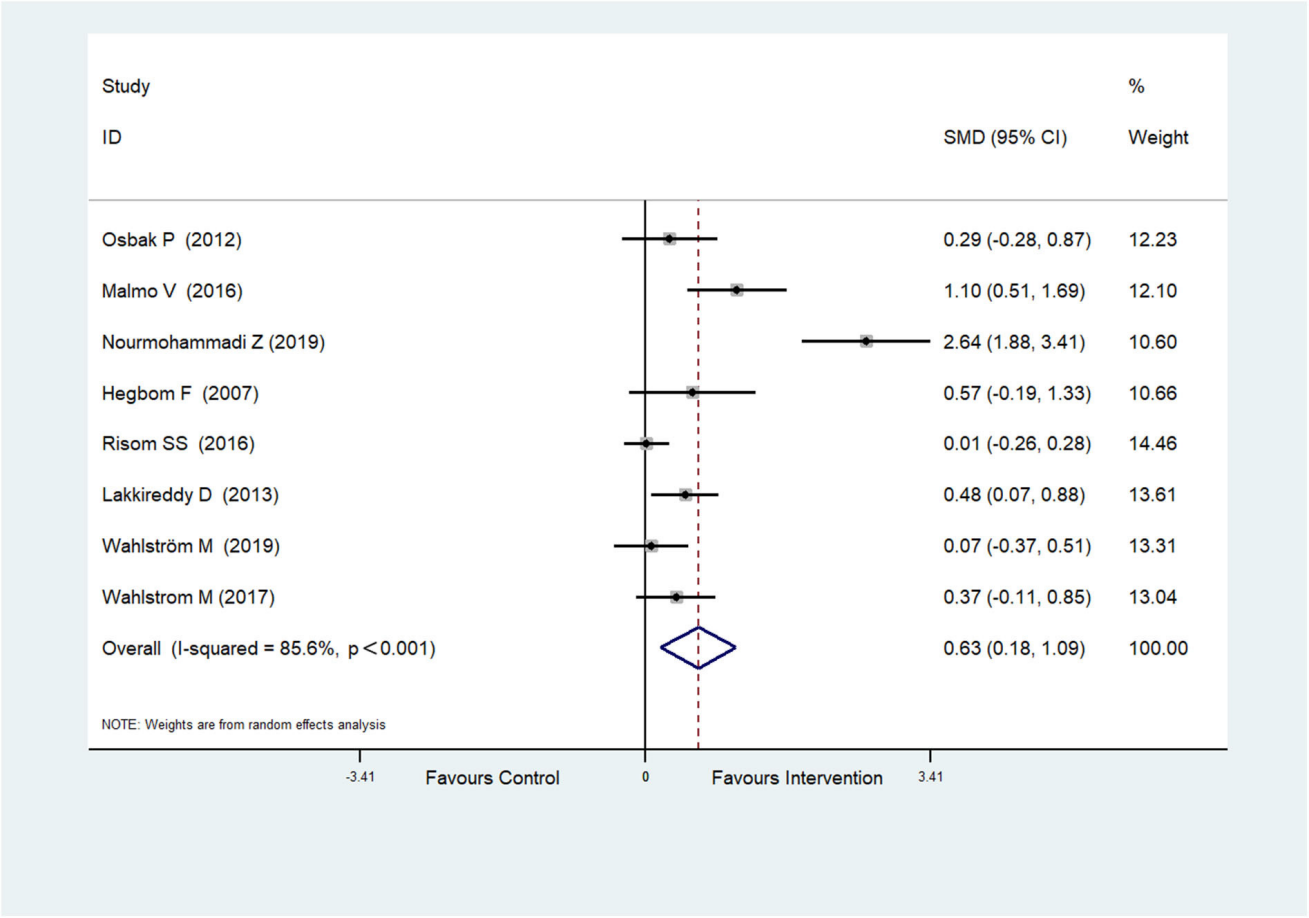
Forest plot shows the ES of physical exercise on Physical Functioning in AF patients.

Eight RCTs (Hegbom et al., 2007; Osbak et al., 2012; Lakkireddy et al., 2013; Malmo et al., 2016; Risom et al., 2016; Wahlstrom et al., 2017; Wahlström et al., 2020; Nourmohammadi et al., 2019) reported scores of physical functioning, which were improved after physical exercise in AF patients (SMD = 0.63, 95%CI: 0.18–1.09; *p* = 0.006) (Figure 3) with high heterogeneity (*I*^2^ = 85.6%, *p* < 0.001).

**Figure 4:**
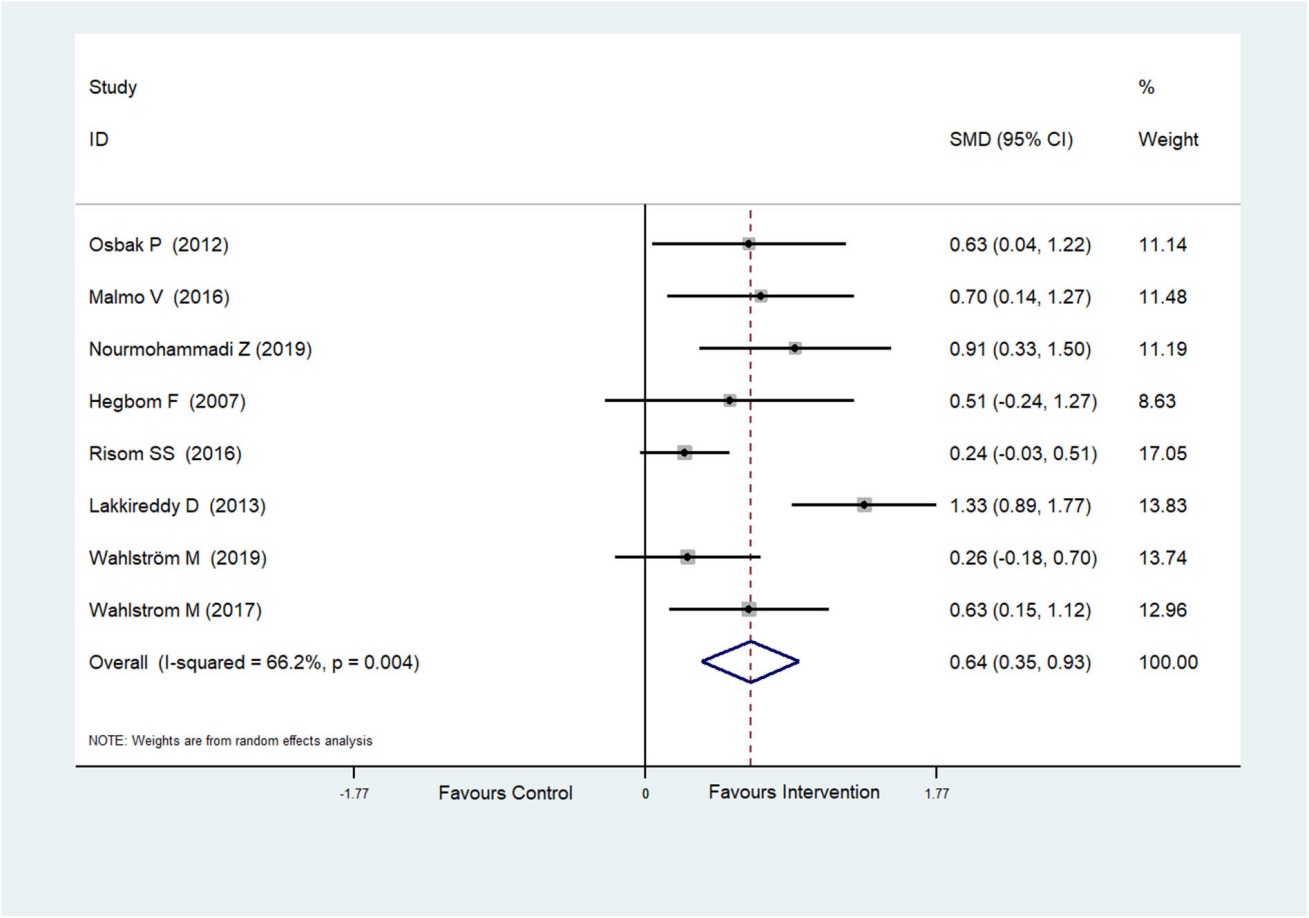
Forest plot shows the ES of physical exercise on General Health Perceptions in AF patients.

Eight RCTs (Hegbom et al., 2007; Osbak et al., 2012; Lakkireddy et al., 2013; Malmo et al., 2016; Risom et al., 2016; Wahlstrom et al., 2017; Wahlström et al., 2020; Nourmohammadi et al., 2019) reported scores of general health perceptions, which were improved after physical exercise in AF patients (SMD = 0.64, 95%CI: 0.35–0.93; *p* < 0.001) (Figure 4) with high heterogeneity (*I*^2^= 66.2%, *p* = 0.004).

**Figure 5:**
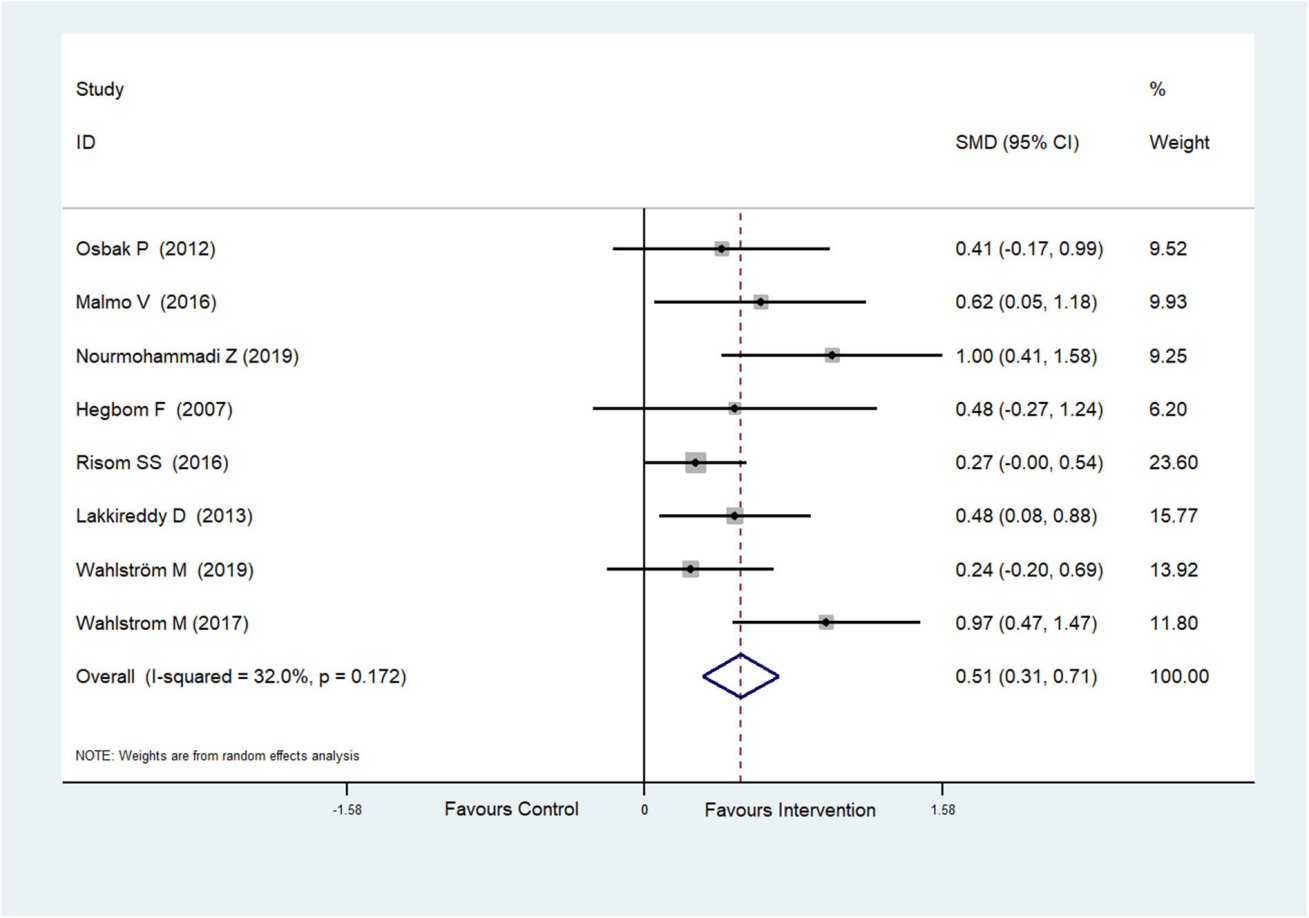
Forest plot shows the ES of physical exercise on Vitality in AF patients.

Eight RCTs (Hegbom et al., 2007; Osbak et al., 2012; Lakkireddy et al., 2013; Malmo et al., 2016; Risom et al., 2016; Wahlstrom et al., 2017; Wahlström et al., 2020; Nourmohammadi et al., 2019) reported scores of vitality, which were improved after physical exercise in AF patients (SMD = 0.51, 95%CI: 0.31–0.71; *p* < 0.001) (Figure 5) with low heterogeneity (*I*^2^= 32.0%, *p* = 0.172).

Because of the high heterogeneity observed in physical functioning (*I*^2^ = 85.6%, *p* < 0.001) and general health perceptions (*I*^2^= 66.2%, *p* = 0.004), we conducted meta-regression to identify the heterogeneity factor from the possible factors (such as the training period, training mode, training duration and exercise intensity) that may cause heterogeneity. The regression results showed that the training mode was the cause of heterogeneity (*p*_physicalfunctioning_ = 0.012, *p*_generalhealthperceptions_ = 0.035). Then, a sub-group analysis was conducted according to the training mode. The result showed that aerobics and Yoga could significantly increase the scores of physical functioning and general health perceptions (Table 4).

**Table 4:** Subgroup analyses of effect size.

	**SMD**	**95%CI**	***P***	***I^**2**^* (%)**	**df**
**PHYSICAL FUNCTIONING**					
Aerobics	1.32	0.07–2.58	**0.038**	91.3	2
Combination training	0.17	−0.32–0.67	0.49	45.2	1
Yoga	0.531	0.06–0.57	**0.015**	0.0	2
**GENERAL HEALTH PERCEPTIONS**					
Aerobics	0.75	0.41–1.08	**0.00**	0.0	2
Combination training	0.18	−0.07–0.44	0.155	0.0	1
Yoga	0.74	0.11–1.38	**0.022**	82.8	2
**6MWT**					
≥60 min	0.88	0.03–1.72	**0.042**	82.5	2
<60 min	0.37	−0.05–0.78	0.082	0.0	1

### Effects of Physical Exercise on Exercise Ability in Patients With AF

**Figure 6:**
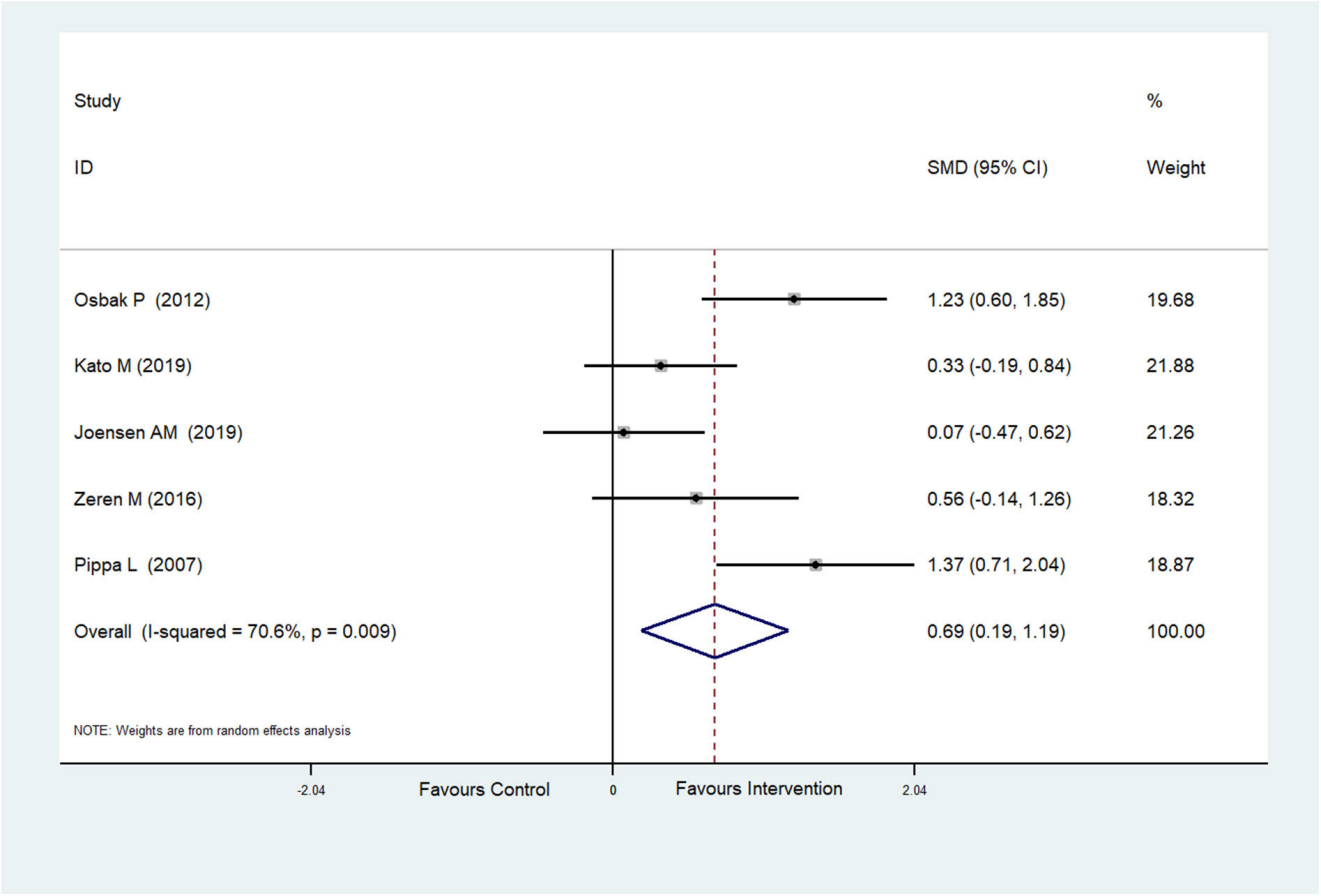
Forest plot shows the ES of physical exercise on 6MWT in AF patients.

The effect of physical exercise on exercise ability was measured by the 6MWT. Five RCTs (Pippa et al., 2007; Osbak et al., 2012; Zeren et al., 2016; Joensen et al., 2019; Kato et al., 2019) reported the results of 6MWTs, which were improved after physical exercise in AF patients (SMD = 0.69, 95%CI: 0.19–1.19; *p* = 0.007) (Figure 6) with high heterogeneity (*I*^2^= 70.6%, *p* = 0.009). After meta-regression analysis, the training duration was selected as the heterogeneity factor (*p* = 0.047) from the possible factors (training period, training mode, training duration and exercise intensity) that may cause heterogeneity. Then sub-group analysis was conducted according to the training duration (≥60 and <60 min). We found that exercise ≥60 min could significantly improve 6MWT performance.

### Effects of Physical Exercise on Cardiopulmonary Fitness in Patients With AF

**Figure 7:**
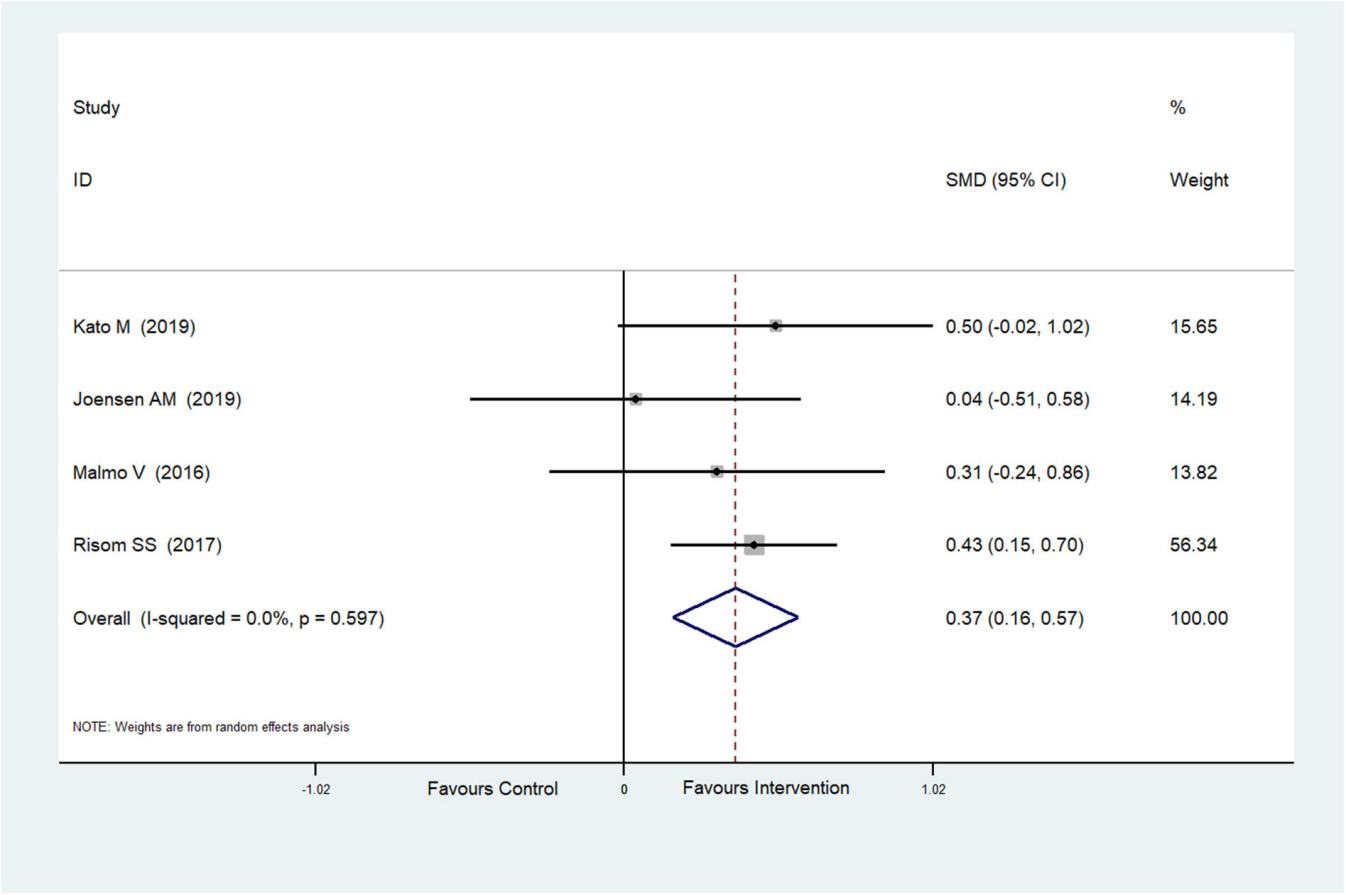
Forest plot shows the ES of physical exercise on peak VO_2_ in AF patients.

**Figure 8:**
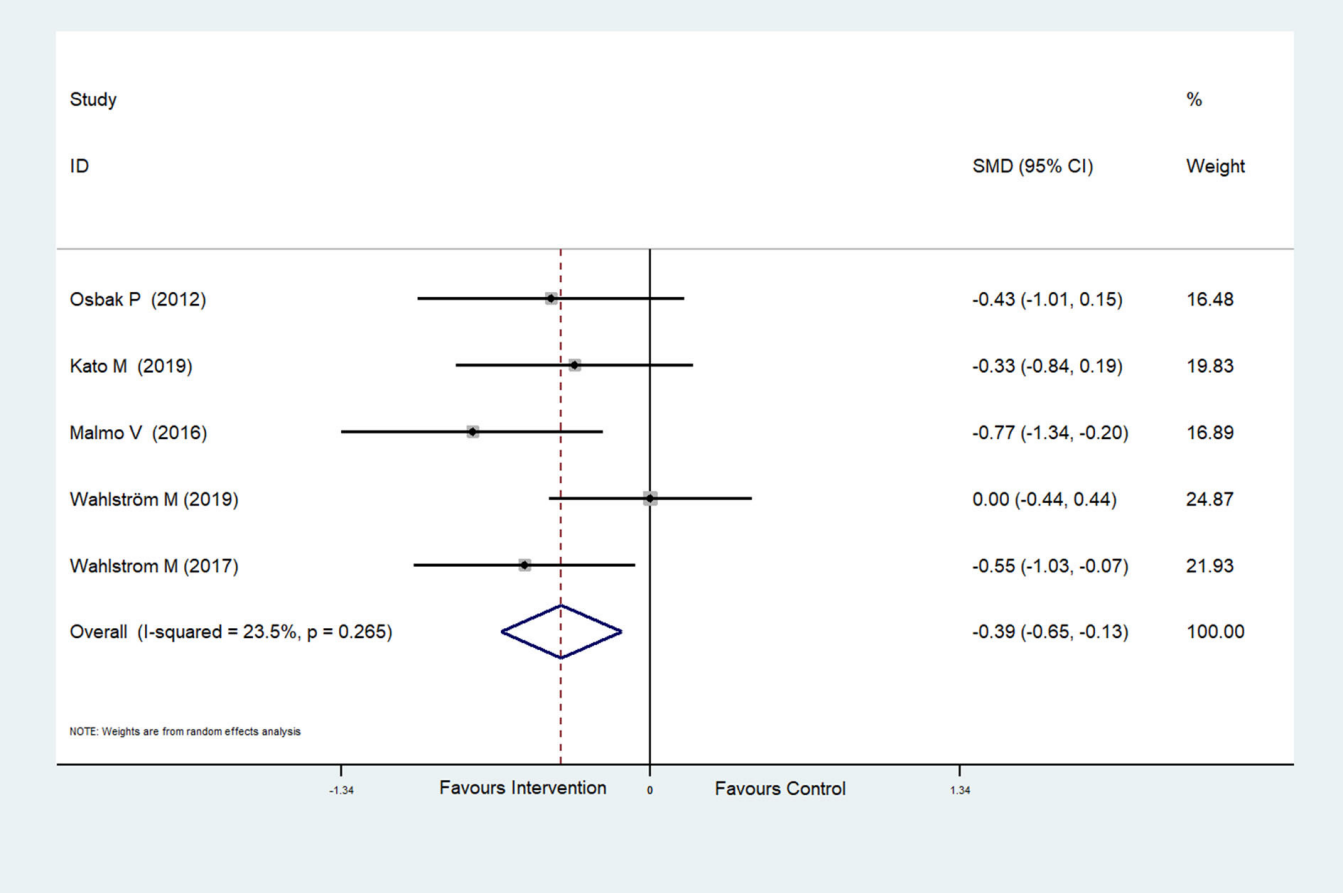
Forest plot shows the ES of physical exercise on resting heart rate in AF patients.

The effect of physical exercise on cardiopulmonary fitness was measured by the peak VO_2_ and resting heart rate. Four RCTs (Malmo et al., 2016; Risom et al., 2017; Joensen et al., 2019; Kato et al., 2019) reported peak VO_2_ values, which were improved after physical exercise in AF patients (SMD = 0.37, 95%CI: 0.16–0.57; *p* < 0.001) (Figure 7) with low heterogeneity (*I*^2^ = 0.0%, *p* = 0.597). Five RCTs (Osbak et al., 2012; Malmo et al., 2016; Wahlstrom et al., 2017; Wahlström et al., 2020; Kato et al., 2019) reported resting heart rates, which were reduced after physical exercise in AF patients (SMD = −0.39, 95%CI: −0.65 to −0.13; *p* = 0.004) (Figure 8) with low heterogeneity (*I*^2^= 23.5%, *p* = 0.265).

### Effects of Physical Exercise on AF Symptoms in Patients With AF

Three RCTs reported AF symptoms (Hegbom et al., 2007; Malmo et al., 2016; Risom et al., 2016) as outcomes. Malmo et al. (2016) measured AF symptoms at the post-intervention follow-up with the Symptom and Severity Checklist questionnaire. They found a larger decrease in the exercise group compared with the no exercise group in AF frequency (exercise 11.5 ± 5.3 vs. no exercise 16.7 ± 5.2), but the authors did not report if the difference was statistically or clinically significant. Hegbom et al. (2007) reported AF symptoms after exercise intervention for the entire population based on the Symptom and Severity Checklist and found no change between baseline and after exercise training in the frequency of AF symptoms (baseline 14 ± 5 vs. after exercise 12 ± 7). In the study of Risom et al. (2016), AF symptoms were reported by 3 patients in the exercise group and 3 patients in the no exercise group. In Hegbom's study, patients served as their own controls, which is an incomplete crossover design study. Therefore, we were unable to conduct a meta-analysis on AF symptoms and only summarized the results here.

### Publication Bias

Begg's test and Egger's test of publication bias were performed for each study. The results showed no publication bias in this meta-analysis (*p* > 0.1) (Table 5). However, because of the small sample size of the included studies, the results of publication bias may not be completely accurate.

**Table 5:** *P* for Begg's/Egger's test.

	***P* for Begg's test**	***P* for Egger's test**
Physical functioning	0.266	0.251
General health perceptions	0.711	0.978
Vitality	0.108	0.374
6MWT	0.462	0.244
peak VO_2_	0.327	0.777
Resting heart rate	0.806	0.296

## Discussion

In the present meta-analysis, we found that (i) physical exercise had a positive effect on the QoL, exercise ability and cardiopulmonary fitness in patients with AF; (ii) the exercise mode could predict the effect of physical exercise on QoL; and (iii) the exercise duration could predict the effect of physical exercise on exercise ability. Moderate exercise is beneficial for AF patients likely because it has anti-adrenalin, anti-inflammatory, anti-ischemic, anti-arrhythmia and anti-thrombosis effects, which can reduce the risk factors of AF (Kato et al., 2019). Because of the limited methodological quality of the included RCTs, the evidence of the benefits of physical exercise for AF patients remains limited. Therefore, there is insufficient data to draw clear conclusions regarding the effect of physical exercise on AF symptoms.

The results of our study are broadly consistent with the previous meta-analysis. Risom et al. (2017) showed that exercise had positive effects on general health perceptions (SMD = 7.11, 95%CI: 3.46–10.77; *p* = 0) and vitality (SMD = 6.1, 95%CI: 1.91–10.3; *p* = 0). As an update and supplement meta-analysis, our study included RCTs studying Yoga and recently published RCTs. In addition, further subgroup analyses were conducted and revealed that training mode and training duration were the variables affecting the QoL and exercise ability of AF patients. To the best of our knowledge, this is the first systematic review and meta-analysis doing this work.

High heterogeneity was associated with the analysis of QoL and exercise ability, so we conducted meta-regression and sub-group analyses to identify the contributing factors. Heterogeneity may arise from several aspects, but the most important part is the training mode and duration. Other possible reasons include the exercise intensity, lack of a standard course of treatment and number of sessions per week, all of which can be quite different. Therefore, heterogeneity was caused by both clinical and methodological sources.

The main symptoms experienced by AF patients are dyspnea, palpitations and decreased exercise tolerance, leading to a reduced QoL. SF-36 is a universal measurement scale for evaluating QoL and includes questionnaires in 8 fields (Patel et al., 2007). The RCTs selected for analysis in our study mainly focused on physical functioning, general health perceptions and vitality. The present study demonstrated that aerobics and Yoga provided significant improvements in QoL compared with small or trivial improvements after combined aerobics and resistance training. Similarly, Ostman et al. (2017) indicated that aerobics alone could improve QoL, while a combination of aerobic and anaerobic exercises or anaerobic ones alone did not. Mandic et al. (2009) compared aerobics with a combination of aerobics and resistance training for CHF patients and reported that aerobics alone improved the QoL of compliant patients. This may be explained by the relationship between aerobic training and the attenuation of neurohormonal responses to triggers of stress (Mohanty et al., 2016). In contrast, anaerobic exercise or a combination of aerobic and anaerobic exercise did not appear to have this effect. In addition, the emotional benefits of aerobic exercise and Yoga, as well as the positive effects of exercise partnerships, diet and lifestyle changes, should not be underestimated.

The 6MWT is mainly used to evaluate the efficacy of treatments on patients with moderate and severe cardiopulmonary disease (Southard and Gallagher, 2013). Despite differences in outcome variables and measurements, there is a good agreement between the results of various studies. Osbak et al. (2012) found that physical exercise improved 6MWT performance by an average of 13% in AF patients. This result is consistent with a study of CHF patients who participated in a 52-weeks exercise training trial and achieved a 10% to 15% improvement in 6MWT performance (Khand et al., 2003). Due to reduced mobility in patients with heart disease, changes in local hemodynamics often occur, further resulting in a loss of skeletal muscle mass and strength (Plisiene et al., 2008; Haykowsky et al., 2009). Muscle mass and strength can be markedly improved by physical training, which enhances exercise ability. As for the exercise duration, American Heart Association recommends moderate-intensity aerobic exercise for about 60 min every day for several months to reduce the risk of cardiovascular disease (Nelson et al., 2007). Compared with the 120-min exercise duration, the 60-min duration is usually accompanied by a higher compliance rate (Conraads et al., 2012; De Maeyer et al., 2013). In fact, only a few patients can achieve long-term compliance >6 months and reach 120 min of exercise each time (De Maeyer et al., 2013).

Cardiopulmonary fitness is improved after physical exercise by enhancing the peak VO_2_ and reducing the resting heart rate. Moreover, a meta-analysis of RCTs found that the peak VO_2_ increased significantly after exercise training in permanent AF patients (Kato et al., 2017). The underlying mechanism may be related to improved cardiac output and peripheral skeletal muscle oxygen uptake (Kato et al., 2017).

To clarify the effect of exercise on the onset of AF, we assessed the effect of physical exercise on AF symptoms. The results of Malmo et al. (2016) support the conclusion that exercise reduces the frequency of AF symptoms. Hegbom et al. (2007) compared before and after the exercise intervention, and there was no statistical difference in symptom frequency, but a significant difference was observed in symptom severity. Risom et al. (2016) only reported the number of patients with exercise-related and exercise-independent AF symptoms. Therefore, due to insufficient evidence and the small sample size, it was not possible to draw a conclusion based on our results.

The current study appears to support the hypothesis that the mechanism by which vigorous exercise increases the risk of AF is different from the mechanism by which moderate exercise reduces the risk, which may be related to the unique characteristics of the studied population (Morseth et al., 2018). Vigorous exercise is always performed by specialized athletes, and they respond to high-intensity exercise through cardiac physiological changes. The mechanism may include the influence of the autonomic nervous system, remodeling of the heart, and trigger zone of the pulmonary veins and atria (Benito et al., 2011; Brugger et al., 2014). The mechanism by which moderate physical exercise reduces the risk of AF may involve a unique approach because physical exercise can potentially reduce the risk of AF through its beneficial effects on cardiovascular risk factors.

## Strengths and Limitations

We believe this is the most comprehensive meta-analysis measuring the effects of physical exercise on AF, including QoL, exercise ability, cardiopulmonary fitness and AF symptoms. Training mode and training duration are the factors influencing physical exercise to improve the QoL and exercise ability of AF patients. There was no evidence of publication bias in this study. However, we recognize that this study has some limitations. First, the majority of included trials were relatively small with short-term follow-ups, and thus the number of reported events was limited. Second, the population of several studies combined paroxysmal AF with persistent AF, and then randomly assigned participants to the exercise group or no-exercise group. As a consequence, we could not completely separate patients with different types of AF. Moreover, some studies included patients with chronic AF and were unable to perform classification, which led to the inability to perform subgroup analysis based on the classification of AF, resulting in a large population difference. In future research, we need to explore the effect of exercise on patients with different types of AF.

## Conclusions

Our meta-analysis shows that physical exercise is an effective method to improve the QoL, exercise ability and cardiopulmonary fitness for AF patients. For this purpose, physicians should consider both the training mode and training duration when designing programs. However, the quality of the original RCTs included in our meta-analysis is limited, and methodological RCTs should also be performed. We should continue to expand our cumulative meta-analysis with future trials to identify safe and well-tolerated treatments that could help AF patients.

## Data Availability Statement

All datasets generated for this study are included in the article/Supplementary Material.

## Author Contributions

ShuqS, JS, and QJ conceived and designed the study. ShuaS and GY performed the database search and extracted the data. ShuqS analyzed the data and wrote the manuscript. QJ edited the English. YH revised the manuscript. All authors contributed to the article and approved the submitted version.

## Conflict of Interest

The authors declare that the research was conducted in the absence of any commercial or financial relationships that could be construed as a potential conflict of interest.
