# Supplementary figures and images for: Efficacy of Physical Exercise on the Quality of Life, Exercise Ability, and Cardiopulmonary Fitness of Patients With Atrial Fibrillation: A Systematic Review and Meta-Analysis
Source: Front Physiol. 2020 Jul 24;11:740. doi: 10.3389/fphys.2020.00740 (PMC7393267; doi:10.3389/fphys.2020.00740)

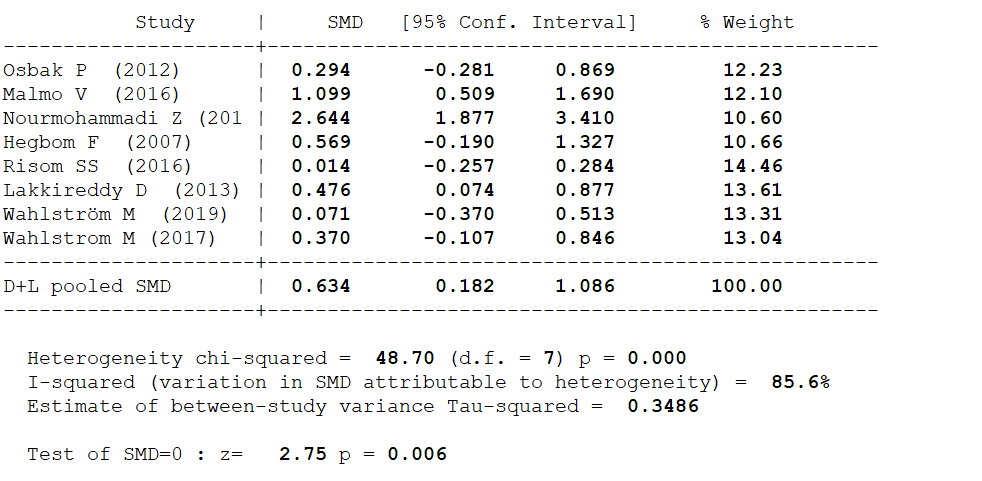

Supplement: Supplementary file 1 [file Image_1.PNG]

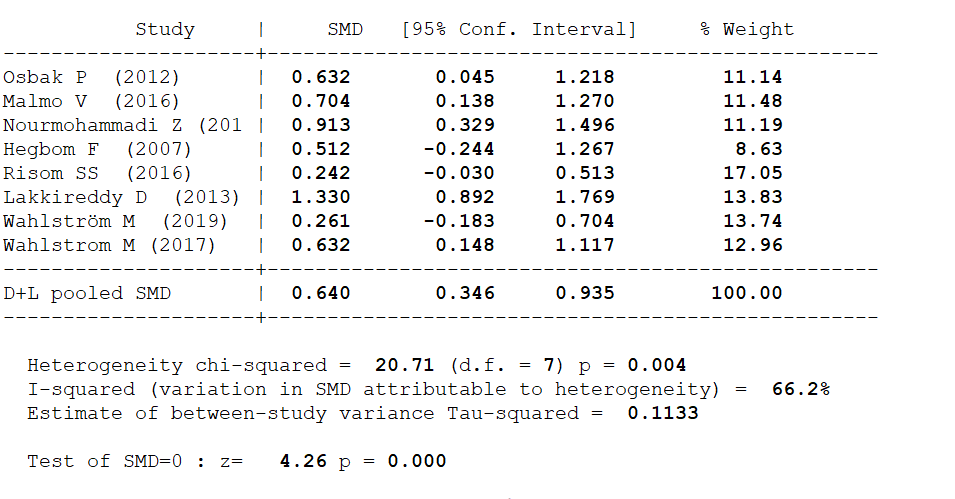

Supplement: Supplementary file 2 [file Image_2.PNG]

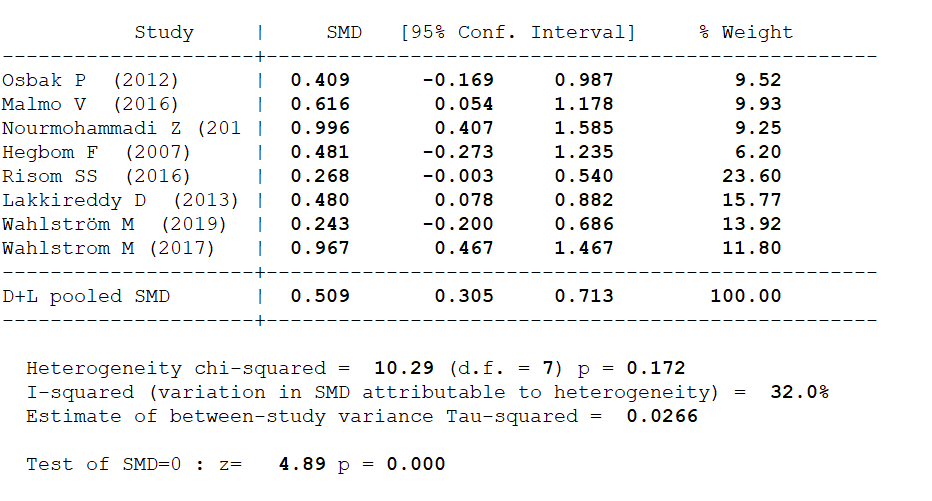

Supplement: Supplementary file 3 [file Image_3.PNG]

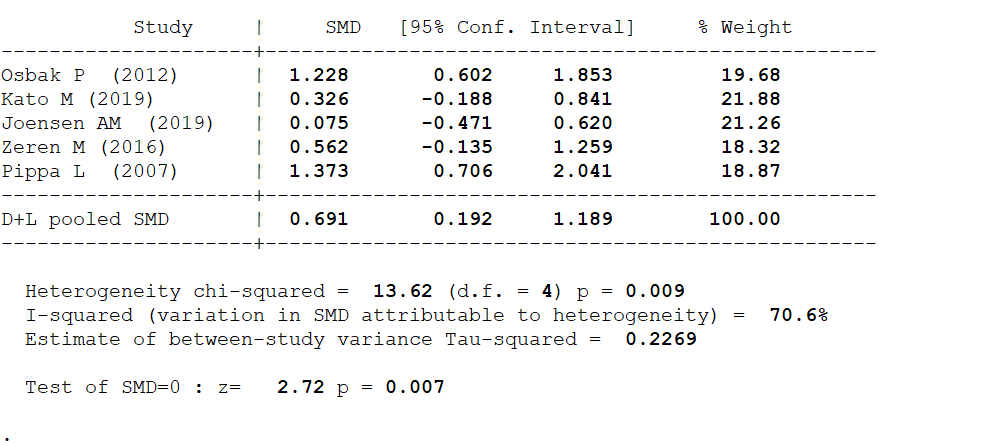

Supplement: Supplementary file 4 [file Image_4.PNG]

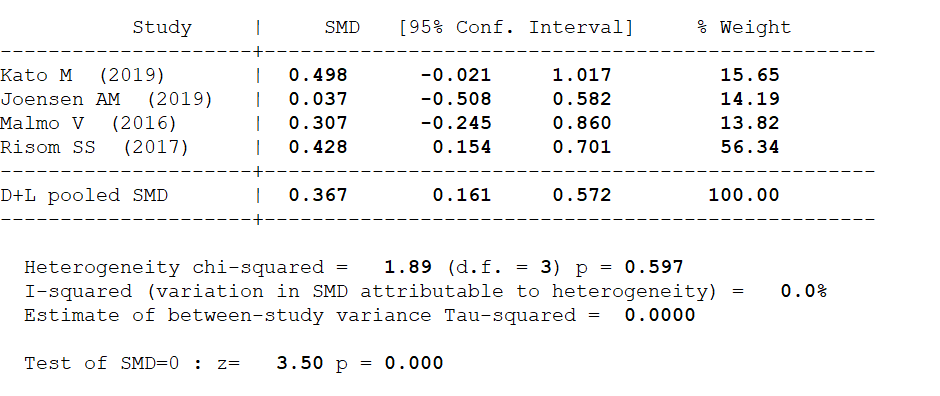

Supplement: Supplementary file 5 [file Image_5.PNG]

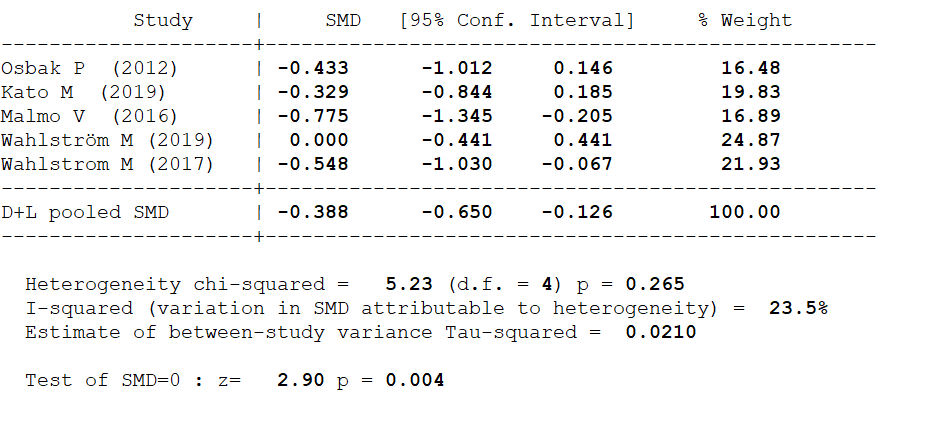

Supplement: Supplementary file 6 [file Image_6.PNG]
